# Gender Differences in Obstructive Sleep Apnea: The Value of Sleep Questionnaires with a Separate Analysis of Cardiovascular Patients

**DOI:** 10.3390/jcm9010130

**Published:** 2020-01-03

**Authors:** Athanasia Pataka, Seraphim Kotoulas, George Kalamaras, Sofia Schiza, Konstantinos Sapalidis, Dimitirios Giannakidis, Nikolaos Michalopoulos, Charilaos Koulouris, Zoi Aidoni, Aikaterini Amaniti, Izoldi Bouloukaki, Evangelos Chatzopoulos, Konstantinos Romanidis, Panagoula Oikonomou, Paschalis Steiropoulos, Georgia Trakada, Anastasios Vagionas, Aris Ioannidis, Iason Nikolaos Katsios, Alexandru Marian Goganau, Pavlos Zarogoulidis, Christoforos Kosmidis

**Affiliations:** 1 Respiratory Failure Unit, G. Papanikolaou Hospital, Aristotle University of Thessaloniki, 57010 Thessaloniki, Greece; patakath@yahoo.gr (A.P.); akiskotoulas@hotmail.com (S.K.); kalamaras.giorgos@gmail.com (G.K.);; 2 Sleep Disorders Unit, Department of Respiratory Medicine, Medical School, University of Crete, 71110 Heraklion, Greece; schiza@med.uoc.gr (S.S.);; 3 3rd Department of Surgery, “AHEPA” University Hospital, Aristotle University of Thessaloniki, Medical School, 54636 Thessaloniki, Greece; sapalidiskonstantinos@gmail.com (K.S.); giannakidis.d@gmail.com (D.G.); nmichalopoulos1@outlook.com (N.M.); charilaoskoulouris@gmail.com (C.K.); dr.ckosmidis@gmail.com (C.K.); 4 Anesthesiology Department, “AHEPA” University Hospital, Aristotle University of Thessaloniki, Medical School, 54636 Thessaloniki, Greece; zoiaidoni@yahoo.com (Z.A.); amanitik@gmail.com (A.A.); 5 Second Department of Surgery, University Hospital of Alexandroupolis, Medical School, Democritus University of Thrace, 68100 Alexandroupolis, Greece; romanidis@yahoo.com (K.R.); pen.ek@hotmail.com (P.O.); 6 Department of Pneumonology, Democritus University of Thrace, 68100 Alexandroupolis, Greece; steiropoulos@yahoo.com; 7 Division of Pulmonology, Department of Clinical Therapeutics, National and Kapodistrian University of Athens School of Medicine, Alexandra Hospital, 11528 Athens, Greece; gtrakada@hotmail.com; 8 Oncology Department, General Hospital of Kavala, 65500 Kavala, Greece; 9 General Surgery Clinic 1, University of Medicine and Pharmacy of Craiova, Craiova County Emergency Hospital, 200009 Craiova, Romania

**Keywords:** gender, sleep apnea, questionnaires, Epworth, Berlin, Stop Bang, Athens Insomnia Scale, Fatigue Scale

## Abstract

Background: Gender affects the clinical presentation of obstructive sleep apnea (OSA). The classic OSA symptoms, such as sleepiness, snoring, and apnea, are not so frequent in women. Objectives: To evaluate possible gender differences in questionnaires used for OSA prediction, such as the Epworth Sleepiness Scale (ESS), STOP, STOP Bang (SB), Berlin Questionnaire (BQ), Athens Insomnia Scale (AIS), and Fatigue Scale (FS). Methods: 350 males were matched with 350 women referred to a sleep clinic, according to OSA severity. All responded to the questionnaires and underwent a sleep study. Cardiovascular disease (CVD) patients were separately analyzed. Results: ESS did not differ between genders. SB was higher in males, whereas STOP, BQ, AIS, and FS were higher in females. BQ presented the highest sensitivity in both genders, whereas STOP exhibited the highest specificity in males and ESS in females. AIS and FS were more sensitive and SB more specific in females, whereas BQ was more specific in males. For severe OSA, the predictive values of SB and BQ were almost similar for both genders; however AIS and FS were higher in women. CVD patients presented higher scores, independent of gender, except for AIS, which was higher in females. Conclusion: Gender-specific evaluation of questionnaires is necessary to prevent OSA under-diagnosis.

## 1. Introduction

Growing evidence suggests that gender affects the incidence and clinical presentation of obstructive sleep apnea (OSA) [1,2,3]. Hormonal influences, the different upper airways shape, body fat distribution, and craniofacial morphology may explain differences between genders. OSA may manifest differently, according to gender. Τhe classic OSA symptoms, such as sleepiness, snoring, and apnea, are reported more frequently in men, whereas fatigue, initial insomnia, depression, and morning headaches are more common in women [2,3,4,5].

OSA symptoms are usually assessed by sleep questionnaires. Several screening tools have been evaluated for identification of the disease [6]. However, it is not clear yet if there is a gender-specific influence in the predictive performance of different questionnaires. OSA has been closely associated with cardiovascular diseases. Possible reasons for this association include the presence of intermittent hypoxia with oxidative stress and inflammation, and the high sympathetic nervous activity, leading to endothelial cell dysfunction, systemic hypertension, and atherosclerosis [7]. Additionally, cardiovascular risk factors such as obesity, type 2 diabetes, and dyslipidemia are frequent OSA co-morbidities [7]. 

The objectives of the present study were to examine the gender differences in commonly used questionnaires for the evaluation of OSA, such as the Epworth Sleepiness Scale (ESS), STOP and Stop Bang (SB), and Berlin Questionnaire (BQ), but also other questionnaires assessing OSA symptoms, such as the Athens Insomnia Scale (AIS) and Fatigue Scale (FS), in males and females matched for their severity of OSA. Additionally, a separate analysis of patients suffering from cardiovascular disease (CVD) was performed to evaluate possible additional differences in this population.

## 2. Methods

A prospective study of patients referred to the Sleep Clinic of the Respiratory Failure Unit of G Papanikolaou Hospital from 2016 to 2018 was conducted. Patients >18 years with symptoms suggestive of OSA, such as snoring, witnessed apneas, daytime sleepiness, fatigue, and insomnia, were included. Patients who refused to participate or suffered from another sleep disorder were excluded. Informed consent was obtained from all participants. We aimed to compare the gender differences in the predictive values of the questionnaires in two groups (350 men and 350 women), matched for the severity of OSA. A separate analysis was performed for the patients suffering from CVD (Coronary disease, stroke > 6 months before the study, not hypertension only) that were included in the matched groups.

The Local Ethics Committee approved the protocol. A general sleep questionnaire of the sleep laboratory (sleep habits, snoring, witnessed apneas, leg movements, and nocturia), Epworth Sleepiness Scale (ESS) [8], STOP and STOP Bang (SB) [9], Berlin Questionnaire (BQ) [10], Athens Insomnia Scale (AIS) [11] and Fatigue Scale (FS) [12] was completed by the participants. The Body Mass Index (BMI), age, neck circumference, and gender were also recorded. Hypertension was defined as a systolic pressure >140 mmHg or diastolic pressure >90 mmHg and/or ongoing antihypertensive medication.

The ESS [8] is a widely used questionnaire for the assessment of excessive daytime sleepiness (EDS). The possibility of dozing off in eight common situations is rated by the patients from 0 to 3. When the score is more than 10, ESS is considered abnormal (scores between 0 and 24). 

The SB [9] is a brief and simple screening questionnaire for assessing the risk for OSA. It consists of eight items: Snoring, tiredness during the daytime, observed apneas, high blood pressure (STOP), BMI > 35 kg/m^2^, age (>50 years old), neck circumference (>40 cm), and gender (male) (BANG). Each positive answer is assigned one point. When ≥2 of the 4 questions of the STOP portion or ≥3 of the 8 questions of the STOP BANG are answered positively, there is a high risk for OSA. 

The BQ [10] is a simple screening questionnaire for assessing OSA risk. It consists of three categories: snoring severity, EDS, and history of hypertension or obesity. If more than two categories are positive, there is a high risk for OSA.

The AIS [11] is a questionnaire developed to evaluate insomnia problems. Each item is rated from 0 (no problem at all), 1 (mild problem), and 2 (marked problem), to 3 (very serious problem). The first five items assess difficulty with sleep induction, awakenings during the night, early morning awakening, total sleep time, and overall sleep quality. The three last items assess the next-day consequences of insomnia, such as problems with one’s sense of wellbeing, functioning, and daytime sleepiness. A cut-off score of ≥6 is used to establish the diagnosis of insomnia.

The FS [12] evaluates fatigue by asking the subjects to express their fatigue from 0 to 10 for the previous two-week period. Scores of 5–6 express mild fatigue, 7–8 moderate fatigue, and 9–10 severe fatigue. In this study, fatigue was defined as an FS score of 5 or higher. 

All the patients underwent a sleep study performed with the Embla Embletta^®^ GOLD Portable Sleep System that involved airflow recording with an oronasal thermistor and nasal pressure transducer, the detection of thoracic and abdominal respiratory effort, the recording of one’s body position, and oximetry. The sleep studies were manually scored according to the American Academy of Sleep Medicine (AASM) guidelines [13]. A drop of airflow ≥90% of the baseline for at least 10 sec was defined as apnea and a decrease in airflow of at least 30% for at least 10 sec with oxygen desaturation of ≥3% from the pre-event baseline was defined as hypopnea. Obstructive events were defined as airflow limitation, but the persistence of respiratory effort. The respiratory event index (REI) was defined as the number of apneas and hypopneas per time in bed and the Oxygen Desaturation Index (ODI3) as the number of oxygen desaturations ≥3% during the time in bed. OSA severity was determined by the REI: mild: REI 5 to 15; moderate: REI greater than 15 to 30; and severe: REI greater than 30. 

### Statistical Analysis

SPSS version 17.0 (SPSS Science, Apache Software Foundation, Chicago, IL, USA) was used. Data were presented as the mean ± SD, unless otherwise stated. Tests were two-tailed and *p* < 0.05 was accepted as statistically significant. To separate parametric from non-parametric variables, normality tests using the Shapiro–Wilk test were performed. To detect significant differences between the two groups, the Independent-Samples *T* Test and the Mann–Whitney *U* test were used for parametric and non-parametric variables, respectively. In order to compare the predictive values of the questionnaires, the sensitivity (Se), specificity (Sp), positive predictive values (PPV), negative predictive values (NPV), and positive and negative likelihood ratios (LR+/LR−) were calculated. In order to compare their predictive values for the two genders, the McNemar x^2^ test was used. The discriminatory ability of each questionnaire was evaluated using receiver operating characteristic (ROC) curves. The area under the curve (AUC) was calculated for REI > 15 events/h. We mainly focused on the performance of the questionnaires at REI > 15, because this is the cut-off where treatment is recommended in our centre. The Pearson correlation coefficient was used for the assessment of possible significant correlations between different variables. The chi-square value was used for categorical variables and Spearman’s correlation coefficient for nonparametric values. Cramer’s V test was used to measure the association between two nominal variables, and the odds ratio (OR) was calculated with separate bivariate logistic regression models. Multiple regression analysis was used for associations of sleep symptoms and sleep questionnaires with OSA (REI > 15), stratified by gender and by cardiovascular disease. Additionally, multiple regression analysis, after adjusting for age, BMI, and smoking, was performed to evaluate the association of cardiovascular co-morbidities with gender and the severity of OSA.

## 3. Results

The main characteristics of the two matched groups (*n* = 350 each) and the differences between genders are presented in Table 1. The ESS did not differ between genders for REI > 15. However, the SB score was higher in males, whereas STOP, BQ, AIS, and FS were higher in female subjects (Table 1). 

The groups were matched for the severity of OSA (for both males and females: no OSA: 28%; mild OSA: 16%; moderate OSA: 21%; severe OSA: 35%). Women were older, with a higher BMI, and hip and waist circumference. Cardiovascular risk factors, such as obesity, hypertension, and type 2 diabetes, were higher in women (Table 2). Dyslipidemia did not differ between genders. However, other co-morbidities, such as arrhythmias, hypothyroidism, and depression, were more common in females (Table 2). When multiple regression analysis was performed, after adjusting for age, BMI, and smoking, to evaluate the association of cardiovascular co-morbidities with gender and the severity of OSA, the only significant association was for hypertension in women suffering from severe OSA (OR: 2.034 (95% CI: 1.05–3.93), *p* = 0.035) (Table 3).

The predictive values of different screening questionnaires for REI > 15 for both genders are presented in Table 4. The BQ presented the highest sensitivity in both genders, whereas the STOP had the highest specificity in males and the ESS in females. STOP presented a low sensitivity in both genders (statistically significantly lower in males) that was improved when the BANG portion of the questionnaire was implemented. There were statistically significant differences between genders concerning the sensitivities of the AIS and the FS (more sensitive in females) and the specificities of SB (more specific in females) and BQ (more specific in males) for REI > 15 (Table 3). The BQ presented the best OR in males, whereas the SB displayed the best OR in women. The SB presented the best AUC in both genders (Table 3). Weak correlations were found between REI and all the questionnaire scores, except for BQ (i.e., for males: ESS: *r* = 0.14, *p* = 0.07; AIS: *r* = 0.1, *p* = 0.25; STOP: *r* = 0.27, *p* = 0.001; SB: *r* = 0.3, *p* = 0.004; FS: *x*^2^ = 2.4, *p* = 0.13; Cramer’s *V* test = 0.09, *p* = 0.08; BQ: *x*^2^ = 32.8, *p* < 0.001; Cramer’s *V* test = 0.3, *p* < 0.001; and for females: ESS: *r* = 0.25, *p* = 0.001; AIS: *r* = 0.05, *p* = 0.5; STOP: *r* = 0.15, *p* = 0.05; SB: *r* = 0.25, *p* = 0.01; FS: *x*^2^ = 6.1, *p* = 0.02; Cramer’s *V* test = 0.13, *p* = 0.02; BQ: *x*^2^ = 13.5, *p* = 0.001; Cramer’s *V* test = 0.2, *p* < 0.001).

When the questionnaire scores were compared according to OSA severity, for mild OSA, SB was statistically higher in males (4 ± 1 vs. 3.3 ± 1.35, *p* = 0.01), whereas BQ was higher in females (*x*^2^ = 5.5, *p* = 0.02); for moderate OSA, AIS was significantly higher in females (♂ 7.56 ± 4.9 vs. ♀ 11.5 ± 5.2, *p* < 0.001) and SB showed a trend of being higher in males (♂4.4 ± 1.2 vs. ♀ 2.8 ± 0.84, *p* = 0.08); and for severe OSA, all questionnaires, apart from SB, were found to be higher in females (ESS: ♂ 9.5 ± 4.7 vs. ♀ 10.8 ± 4.7, *p* = 0.03; BQ: *x*^2^ = 17.2, *p* = 0.001; AIS: ♂ 7.8 ± 5 vs. ♀ 10.2 ± 5.8, *p* = 0.05; STOP: ♂ 2.4 ± 0.84 vs. ♀ 2.83 ± 0.8, *p* < 0.001; FS: *x*^2^ = 5.26, *p* = 0.02). 

**Figure 1:**
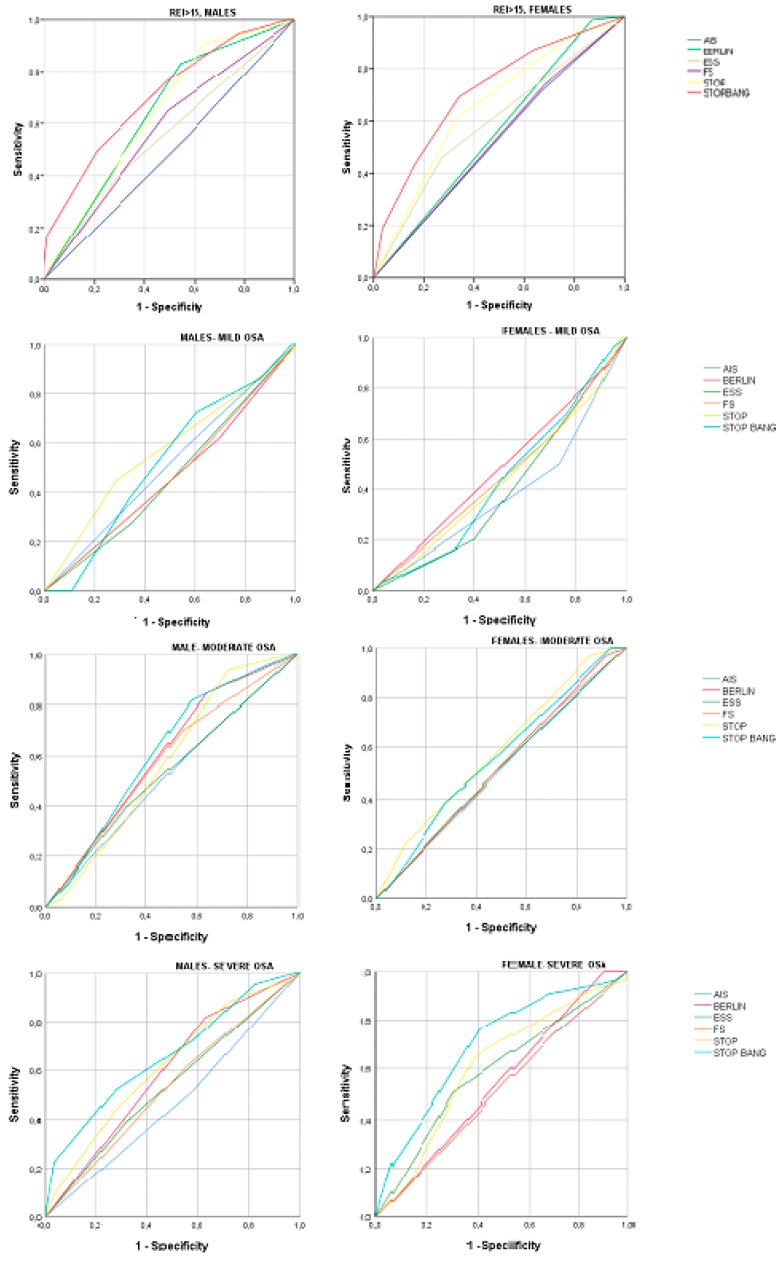
Area under the curve (AUC) of receiver operating characteristic (ROC) curves for the discriminatory ability of each questionnaire in both genders in the different severity groups of OSA (for REI > 15, mild, moderate, and severe OSA). ESS = Epworth Sleepiness Scale, BERLIN = Berlin Questionnaire, AIS = Athens Insomnia Scale, and FS = Fatigue Scale.

When the predictive values of both groups were analyzed according to the severity of OSA, they improved in all questionnaires in severe OSA compared to mild disease. STOP was statistically significantly more sensitive in women with severe OSA (♂ 44.1% vs. ♀70.1%) compared with men, whereas SB presented the highest sensitivity in men with mild OSA (♂ 74.3% vs. ♀ 43.5%) and the highest specificity in females with severe OSA (♂ 39% vs. ♀ 59.7%). BQ was statistically significantly more sensitive in women with mild disease (♂ 60% vs. ♀ 80.7%) and more specific in men in all OSA severity groups. Additionally, AIS presented a significantly higher specificity in males with mild OSA (♂ 47.7% vs. ♀ 26.5%) and higher sensitivity in females with moderate (♂ 56.3% vs. ♀ 78.2%) and severe (♂ 50% vs. ♀ 72.8%) OSA. FS was significantly more sensitive in women with severe OSA (♂ 57.5% vs. ♀ 71.6%) compared with men in the other severity groups. In mild OSA, STOP presented the highest AUC in males (0.57, 95% CI 0.45–0.69), whereas the BQ exhibited the highest AUC (0.48, 95% CI 0.37–0.6) in females. In moderate OSA, SB presented the highest AUC in males (0.62, 95% CI 0.52–0.72), whereas STOP presented the highest AUC in females (0.59, 95% CI 0.48–0.7). In severe OSA, SB presented the highest AUC in both males (0.66, 95% CI 0.56–0.76) and females (0.7, 95% CI 0.62–0.79) (Figure 1).

When OSA symptoms were analyzed, male patients presented with the ‘more classic’ OSA symptoms, like witnessed apneas. Females more frequently reported symptoms such as fatigue and headaches in the morning, nocturnal awakenings, nocturia, nightmares, memory loss, and attention problems. The differences in sleep apnea symptoms between genders are presented in Table 5. 

In a separate analysis, we evaluated the differences between patients suffering from CVD (*n* = 98, 53 men and 45 females, *p* = 0.54) and those who not suffering from CVD (Table 6). Except for ESS and FS, all the other sleep questionnaire scores were higher in patients with CVD. When the above analysis was conducted according to gender, the results were similar to those of Table 1 for patients without CVD. Gender differences between patients with CVD included a higher BMI, waist circumference, and AIS in women, without the significant differences in STOP, SB, or BQ that were found in the group without CVD. 

Multiple regression analyses for associations of sleep symptoms and sleep questionnaires with OSA (REI > 15) stratified by gender and cardiovascular disease were performed (Table 7). For patients with CVD, the presence of witnessed apneas, SB, and FS > 5 was associated with OSA after adjusting for age, gender, BMI, smoking, and alcohol.

## 4. Discussion

In this study, we found that there were gender differences in the predictive values of different questionnaires. Our findings suggest that gender-specific consideration should be incorporated into the application, analysis, and interpretation of sleep questionnaires. It is well-known from previous studies that OSA symptoms differ between genders, with the more classic symptoms reported by males [4,5,6,14,15]. Women with OSA may be misdiagnosed and treated for other diseases, such as depression, insomnia, and hypothyroidism [15], because they present with atypical symptoms, such as fatigue, sleep onset insomnia, or morning headaches [2,3,5,15]. Compared with men with a similar OSA severity, women were found to be heavier users of health care resources because of their atypical symptoms, their poor perception of health, and the overuse of psychoactive medication [16]. 

Sleep questionnaires are frequently used to predict OSA and their predictive performance has been evaluated in the general population and in populations referred to sleep clinics [5,6,17]. In a previous study, we evaluated, for 1850 patients visiting a sleep clinic, the predictive values of sleep questionnaires [18]. In that study, we found that the SB had the highest sensitivity, NPV, AUC, and OR, and when we combined different questionnaires, there was no significant improvement in the predictive values. Similar to our findings, Silva et al. [19] evaluated ESS, STOP, and SB in the general population and found that the SB had the highest sensitivity and that ESS was not as effective for predicting OSA as STOP and SB. Additionally, in a study conducted in India that evaluated nine screening questionnaires [20], the sleep apnea clinical score (SACS) and the SB demonstrated the best positive and negative likelihood ratios, respectively, for OSA prediction. In a systematic review [17], the Wisconsin and BQ were found to have the highest sensitivity and specificity, but no definite conclusion was made regarding the most accurate questionnaire for the prediction of OSA. In another meta-analysis [21], the STOP, SB, BQ, the American Society of Anesthesiologists (ASA) checklist, the Sleep Questionnaire, and the sleep disorders questionnaire (SDQ) were evaluated. BQ and SDQ were the most accurate for screening OSA, whereas ESS was the least accurate [21]. 

However, there are not many studies assessing the gender-specific performance of sleep questionnaires, in particular, in CVD patients. In the study of Westlake et al. [22], the predictive performances of the BQ and SB were assessed in diabetic patients. The analysis included gender differences of the questionnaires and revealed that the predictive performance of BQ and STOP was not affected by gender. However, the sensitivity and specificity of SB were affected, as the SB is the only questionnaire that includes gender as an item. In a recent study, Mou et al. [23] found that SB presented a low specificity in males using the cut off ≥3 and suggested alternative SB scoring systems using optimal operating points for female BMI and male neck circumference, in order to improve its predictive performance. In another study [24,25], the addition of gender in the BQ improved its performance for determining moderate to severe OSA in a sleep clinic. Additionally, in patients scheduled for bariatric surgery [5], with the exception of the ESS, SB and FS predicted OSA better in women than in men.

In our study, STOP and AIS sensitivities and SB and BQ specificities were affected by gender. STOP presented a higher sensitivity in women, but higher specificity in males; however, when the BANG portion was added (i.e., STOP-BANG), the difference became non-significant for the sensitivity, whereas specificity became statistically significantly higher in females. This may be explained by the different clinical presentation between genders, even with the same severity of OSA, with males presenting more frequently witnessed apneas and a larger neck circumference and females exhibiting an older age, higher BMI, hypertension, and tiredness (Table 1 and Table 5) [2,3,5,14,15]. When the predictive performance was evaluated according to the severity of OSA, in severe disease, SB and BQ presented the highest values in both genders (sensitivities SB (♂ 77.6% vs. ♀ 79.1%) and BQ (♂ 83.6% vs. ♀ 99.1%) and higher AUC (Figure 1)). 

In our study, females with OSA presented a higher proportion of hypertension than males, as in previous studies [2,26] However, there are other studies evaluating the effect of gender on the risk of hypertension in OSA with inconsistent results [27,28]. Questionnaires, such as the SB and BQ, assess blood pressure/hypertension as one of their items and differences in blood pressure/hypertension in the population studied may affect their predictive values. In our study, the OR for hypertension was only significant in women with severe disease, after adjusting for age, BMI, and smoking. In other studies, this correlation only existed in male subjects [26,28]. This could be attributed to the characteristics of the population studied. Different body fat distributions and hormonal influences, such as menopause, are possible reasons for gender differences in the relation between hypertension and OSA. In our study, 67% of women with OSA were postmenopausal (none received hormonal therapy) and of them, 70% were hypertensive. 

Women may not manifest the classical symptoms of OSA and are less likely to be diagnosed and treated for OSA compared with men [29,30]. Classical OSA symptoms, such as snoring and apneas, may be underreported by women as they usually present in clinical interviews alone. EDS is not a common finding in women compared with men and the ESS has been found to be a more sensitive measure of EDS in males [31]. In our study, we did not find significant gender differences in the ESS, even after examining different OSA severity groups, possibly because we evaluated patients visiting a sleep clinic. In recent studies [2,4], females with OSA have more frequently presented insomnia symptoms, such as fatigue, headaches, mood disturbances, and impaired memory, similar to our study. The classic OSA symptoms are incorporated in questionnaires, such as SB and BQ, but the ‘nonspecific’ OSA symptoms, such as fatigue (FS), insomnia (AIS), and memory loss, are not usually assessed. Insomnia is considered a predictor of OSA, independent of BMI and EDS [32]. The AIS evaluates insomnia symptoms and in the current study, it has been found to be more sensitive in female patients with OSA, in accordance with previous studies [2,3,4,14,15,30]. The FS evaluates fatigue and was higher in women. Additionally, women with OSA suffer more commonly from depression that is closely associated with both insomnia and fatigue [2,30].

Obesity is a risk factor for the development of OSA and also a major cardiovascular risk factor. Women with OSA have been found to be more obese than men with a similar OSA severity [2,4,5,30]. In the population of our study that was matched for OSA severity, the BMI differed between genders, with women being more obese in all severity groups. The consequences of OSA may differ between genders. Recent data suggest that women suffering from OSA may have greater impairment of quality of life than men [16,30]. 

OSA has been associated with several cardiovascular consequences, such as hypertension, stroke, and arrhythmias, and in some studies, there is an increased incidence of these cardiovascular outcomes in women suffering from severe disease [33]. CPAP and the Mandibular Advancement Device reversed this risk (especially blood pressure) in women with adequate compliance [26,30,34]. An increased awareness of gender differences leading to improved diagnosis and treatment are essential. In the current study, some questionnaires, such as the BQ, were found to be highly sensitive in both genders, whereas others, like the STOP, SB, and the AIS, presented significant gender differences in their predictive values. For severe OSA, the predictive values of SB and BQ were almost similar for both genders; however, AIS and FS were higher in women.

Sleep is a major component of good health and poor sleep, both in terms of duration and quality, has been associated with an increased risk of CVD, diabetes, and hypertension [35]. In a recent study on women, insomnia, snoring, and a high OSA risk were associated with a higher odds of having poor cardiovascular health [36]. In this study, we assessed the possible gender differences of sleep questionnaires in CVD patients. In our analysis, the scores of all the sleep questionnaires were high in the group of CVD patients, especially STOP, SB, and BQ, as they include hypertension and BMI in their items, which were higher in this population. Additionally, FS was higher, suggesting that ‘fatigue’ is an important symptom of OSA in these patients. In the separate gender analysis of this group of patients, no significant differences were observed compared to the results of the whole group, apart from the absence of differences in STOP, SB, or BQ found in the group without CVD.

The main limitation of the present study is the referral bias, as the population studied was referred to a sleep clinic and was not representative of the general population. It would be interesting to study the performance of sleep questionnaires between genders in random patients eligible for OSA screening in a primary care setting. However, we matched the two groups according to OSA severity, as we aimed to evaluate the different questionnaires for the same severity of the disease in both genders. Additionally, our patients underwent limited sleep studies without an electroencephalogram for sleep staging and this could have led to the under-diagnosis of mild OSA. However, in our centre, treatment is offered to moderate to severe OSA (REI > 15) and for that reason, REI > 15 was mainly evaluated. Additionally, questionnaire screening values may vary according to BMI, especially for those that include BMI as a component, such as the SB and the BQ; for that reason, physicians should evaluate them according to the population studied [37].

## 5. Conclusions

In conclusion, knowledge on gender-related differences may improve OSA diagnosis, as women are frequently evaluated and treated according to male criteria. For severe OSA, the predictive values of AIS and FS were higher in women, supporting OSA’s different clinical presentation in women with fatigue and insomnia [1,2,3,4,5,6,7,14,15]. CVD patients presented higher scores in questionnaires independent of gender, except for AIS, which was higher in females.

## Figures and Tables

**Table 1:** Characteristics of the population and differences between genders.

	Males	Females	*P* Value
Age (years)	50.7 ± 14.5	55.5 ± 13.2	<0.001
BMI (kg/m^2^)	26.5 ± 5.55	32.7 ± 10.7	<0.001
Waist circumference (cm)	98.1 ± 13.6	105.9 ± 18.8	<0.001
Hip circumference (cm)	102.6 ± 7.7	115 ± 15.7	<0.001
Neck circumference (cm)	40.6 ± 11.2	37.7 ± 10	0.003
Systolic Blood Pressure (mmHg)	123.3 ± 14.1	125.1 ± 17.2	0.3
Diastolic Blood Pressure (mmHg)	76.5 ± 9.3	75.6 ± 10	0.4
Smoking (pack/years)	27.5 ± 20.7	19.2 ± 12.7	<0.001
REI (events/h)	24.3 ± 23.2	25.1 ± 24.2	0.7
Average Apneas/hypopneas Duration (s)	21.3 ± 8.4	19.6 ± 8.2	0.02
ODI	24.1 ± 23.6	25.2 ± 26.3	0.3
ESS	8.8 ± 4.4	9.34 ± 4.9	0.1
STOP	2.16 ± 0.87	2.47 ± 0.97	<0.001
STOP-BANG	4.1 ± 1.3	3.66 ± 1.5	0.002
Berlin Questionnaire High Risk	70%	87.3%	<0.001
Athens Insomnia Scale	7.46 ± 5.04	10.3 ± 5.8	<0.001
Fatigue Scale > 5	55.6%	64.5%	0.02

BMI = Body Mass Index, REI = respiratory event index, ODI = Oxygen Desaturation Index, and ESS = Epworth Sleepiness Scale.

**Table 2:** Differences between genders concerning co-morbidities.

Co-morbidities	Males	Females	*P* Value
Hypertension	38%	52.1%	<0.001
Arrhythmias	9%	19.6%	0.001
Coronary disease	8.2%	7%	0.1
Stroke	7.1%	6.8%	0.3
Diabetes	10%	17%	0.01
Dyslipidemia	32%	34.8%	0.35
Lung disease	7.7%	11.7%	0.11
Hypothyroidism	1.1%	24.2%	<0.001
Depression	5.4%	10.7%	0.002

**Table 3:** Multiple regression analysis, after adjusting for age, BMI, and smoking, for the association of cardiovascular co-morbidities with gender and the severity of obstructive sleep apnea (OSA).

	Males			Females		
	Mild OR (95%CI) *P* Value	Moderate OR (95%CI) *P* Value	Severe OR (95%CI) *P* Value	Mild OR (95%CI) *P* Value	Moderate OR (95%CI) *P* Value	Severe OR (95%CI) *P* Value
**Hypertension**	1.1 (0.4–3.5) 0.66	0.8 (0.3–2.15) 0.67	1.34 (0.57–3.18) 0.5	1.44 (0.6–3.18) 0.37	1.66 (0.8–3.45) 0.17	2.034 (1.05–3.9) 0.035
**Arrhythmias**	0.99 (0.2–3.8) 0.9	1.48 (0.3–6.4) 0.6	2.3 (0.6–8.4) 0.2	0.76 (0.3–1.9) 0.5	0.8 (0.3–1.9) 0.6	1.03 (0.4–2.7) 0.9
**Coronary disease**	1.8 (0.25–12) 0.55	1.99 (0.3–11.8) 0.44	1.7 (0.3–9.3) 0.54	6.3 (0.66–61.4) 0.1	6.8 (0.8–59) 0.08	5.8 (0.67–48) 0.1
**Stroke**	0.9 (0.85–1) 0.3	1.1 (0.8–8.8) 0.6	3.2 (0.33–31) 0.3	0.7 (0.1–12.3) 0.8	1.01 (0.06–17.3) 0.9	3.69 (0.3–44.1) 0.3

OR = Odds Ratio and CI = confidential interval.

**Table 4:** Differences between genders for the predictive values of different screening questionnaires (for REI > 15).

		Se%	Sp%	PPV%	NPV%	LR(+)/(−)	AUC (95% CI)	OR (95% CI)
**ESS**	**♂**	41.1	75.8	68.1	50.6	1.7/0.78	0.55 (0.46–0.64)	2.19 (1.37–3.5)
	♀	45	70.3	65.15	50.9	1.52/0.78	0.59 (0.51–0.68)	1.94 (1.24–3.035)
**STOP**	♂	43.5 *	79	72.4	52.6	2.08/0.71	0.65 (0.56–0.73)	2.9 (1.8–4.72)
	♀	**61.7**	65.8	69	58.1	1.8/0.58	0.66 (0.57–0.74)	3.1 (1.98–4.84)
**STOP BANG**	♂	78.2	47.3 *	63.7	64.7	1.48/0.46	0.7 (0.63–0.79)	3.22 (1.75–5.9)
	♀	71.9	**67.3**	69.3	70	2.2/0.41	0.715 (0.64–0.79)	5.28 (2.93–9.51)
**Berlin**	♂	82.6	46.4 *	65.7	68.2	1.54/0.37	0.64 (0.56–0.73)	4.12 (2.52–6.74)
	♀	93.4	**20**	58.8	71.4	1.17/0.33	0.56 (0.47–0.645)	3.56 (1.75–7.24)
**AIS**	♂	52.6 *	48.9	50.3	51.1	1.02/0.96	0.49 (0.4–0.58)	1.06 (0.66–1.7)
	♀	**74.8**	35.5	57.9	54.3	1.16/0.99	0.52 (0.44–0.61)	1.6 (0.97–2.76)
**Fatigue Scale**	♂	59.3	50	60.2	49	1.18/0.81	0.58 (0.49–0.67)	1.46 (0.95–2.23)
	♀	70.1	41.9	59.8	53.3	1.21/0.71	0.53 (0.44–0.62)	1.7 (1.09–2.65)

REI = respiratory event index, Se = sensitivity, Sp = specificity, PPV = Positive Predictive Value, NPV = Negative Predictive Value, LR = Likelihood Ratio, AUC = Area Under the Curve, OR = Odds Ratio, ESS = Epworth Sleepiness Scale, AIS = Athens Insomnia Scale, and * difference between genders *p* < 0.05.

**Table 5:** Differences in sleep apnea symptoms * between genders.

	Males	Females	*P* Value
Witnessed Apneas	70%	60.6%	**0.008**
Snoring	93.1%	91.8%	0.5
Tiredness	28%	45.5%	**<0.001**
Headaches	26.1%	43.5%	**<0.001**
Nightmares	22%	39.6%	**<0.001**
Bad mood in the morning	52.3%	59%	0.17
Memory loss	46%	58.9%	**0.001**
Attention problem	12.5%	32.2%	**<0.001**
Nocturia	67.9%	81.5%	0.001
Nocturnal awakening	68.4%	84.6%	**<0.001**

* more than 1 day/week.

**Table 6:** Differences between the characteristics of patients with/without cardiovascular disease.

	Without (*N* = 602)	With (*N* = 98)	*p*
Age (years)	**51.7 ± 13.8**	**65.4 ± 10**	**0.001**
BMI (kg/m^2^)	29.3 ± 9.36	29.6 ± 9.3	0.7
Waist circumference (cm)	101.7 ± 17	104.3 ± 14.7	0.38
Neck circumference (cm)	39.2 ± 11	38.1 ± 4.4	0.5
Smoking (pack/years)	23 ± 13.9	31.2 ± 23	0.09
Hypertension	**33.5%**	**71.6%**	**<0.001**
REI (events/h)	24.3 ± 23.1	28.5 ± 18.9	0.09
ODI	**26.2 ± 24.8**	**30.2 ± 20.4**	**0.05**
ESS	9.1 ± 4.6	8.6 ± 4.9	0.4
STOP	**2.2 ± 0.9**	**2.8 ± 1**	**<0.001**
STOP-BANG	**3.77 ± 1.37**	**4.9 ± 1.5**	**<0.001**
Berlin Questionnaire High Risk	**77%**	**87.8%**	**0.045**
Athens Insomnia Scale	**8.7 ± 5.5**	**10.1 ± 4.04**	**0.04**
Fatigue Scale > 5	60.2	58.2	0.78

BMI = Body Mass Index, REI = respiratory event index, ODI = Oxygen Desaturation Index, and ESS = Epworth Sleepiness Scale.

**Table 7:** Multiple regression analysis * for the associations of sleep symptoms and sleep questionnaires with OSA (REI > 15) stratified by gender and cardiovascular disease.

	Presence of Cardiovascular Disease				Gender			
	No		Yes		Male		Female	
	OR (CI 95%)	*p*	OR (CI 95%)	*p*	OR (CI 95%)	*p*	OR (CI 95%)	*p*
Snoring (yes/no)	1.8 (0.86–3.75)	0.1	**3.8** **(1.36–9.75)**	**0.01**	**3.02** **(1.06–8.6)**	**0.04**	1.53 (0.58–3.98)	0.4
Apneas (yes/no)	**3.004** **(2.05–4.41)**	**<0.001**	**3.001** **(2.01–4.8)**	**0.001**	**3.3** **(1.9–5.77)**	**<0.001**	**2.32** **(1.4–3.87)**	**0.001**
ESS	**1.1** **(1.04–1.17)**	**0.002**	1.07 (0.8–1.39)	0.5	1.06 (0.97–1.16)	0.18	**1.17** **(0.06–1.28)**	**0.001**
Berlin	**3.79** **(1.44–10.0)**	**0.007**	1.8 (0.1–13.3)	0.8	**3.66** **(1.05–12.7)**	**0.04**	5.5 (0.19–15)	0.3
STOP	1.58 (0.8–3.07)	0.17	**1.02** **(0.7–2.4)**	0.4	0.78 (0.29–1.8)	0.5	3.2 (1.16–8.9)	0.025
STOP-BANG	0.8 (0.5–1.41)	0.52	**3.58** **(1.22–9.8)**	**0.02**	1.47 (0.7–3.1)	0.3	0.5 (0.26–1.14)	0.11
AIS	**0.93** **(0.88–0.99)**	**0.025**	0.9 (0.61–1.31)	0.6	0.92 (0.83–1.01)	0.08	**0.93** **(0.82–0.99)**	**0.04**
FS5	1.24 (0.59–2.6)	0.56	**3.2** **(1.13–7.3)**	**0.04**	2.13 (0.63–7.2)	0.22	1.19 (0.4–3.27)	0.7

REI = respiratory event index, OR = odds ratio, CI = confidential interval, ESS = Epworth Sleepiness Scale, AIS = Athens Insomnia Scale, FS5 = Fatigue Scale > 5. * adjusted for age, BMI, gender in the case of cardiovascular disease, smoking, and alcohol.
